# Benchmarking the driver acceleration impact on vehicle energy consumption and CO_2_ emissions^☆^

**DOI:** 10.1016/j.trd.2022.103282

**Published:** 2022-06

**Authors:** Jaime Suarez, Michail Makridis, Aikaterini Anesiadou, Dimitrios Komnos, Biagio Ciuffo, Georgios Fontaras

**Affiliations:** a European Commission, Joint Research Centre (JRC), Ispra, Italy; b ETH Zürich, Institute for Transport Planning and Systems (IVT), Zürich, Switzerland; c FINCONS Group, 20871 Vimercate, Italy

**Keywords:** Driver heterogeneity, Driver characterization, Driving style, Energy consumption, WLTC, CO_2_ emissions

## Abstract

The study proposes a methodology for quantifying the impact of real-world heterogeneous driving behavior on vehicle energy consumption, linking instantaneous acceleration heterogeneity and CO_2_ emissions. Data recorded from 20 different drivers under real driving are benchmarked against the Worldwide Harmonized Light Vehicle Test Cycle (WLTC), first by correlating the speed cycle with individual driver behavior and then by quantifying the CO_2_ emissions and consumption. The vehicle-Independent Driving Style metric (IDS) is used to quantify acceleration dynamicity, introducing driving style stochasticity by means of probability distribution functions. Results show that the WLTC cycle assumes a relatively smooth acceleration style compared to the observed ones. The method successfully associates acceleration dynamicity to CO_2_ emissions. We observe a 5% difference in the CO_2_ emissions between the most favourable and the least favourable case. The intra-driver variance reached 3%, while the inter-driver variance is below 2%. The approach can be used for quantifying the driving style induced emissions divergence.

## 1 Introduction

Road transport is responsible for approximately one-fifth of the global carbon dioxide emissions (CO_2_) in the EU, with passenger road vehicles standing as the main source (45.1%) (Action, 2016, Ritchie, 2020). The European Union (EU) has committed to ambitious reductions in carbon emissions from the transport sector. Nevertheless, the effectiveness of implemented measures depends on the reliability of the vehicle fleet carbon emission values. During the past decades, fuel consumption and CO_2_ emissions of light-duty vehicles (LDVs) have consistently showed an apparent disparity between real-world and official certification values (Weiss et al., 2011) (Fontaras et al., 2017) (Pavlovic et al., 2018). Despite the implementation in 2017 of the Worldwide Harmonized Light Vehicles Test Procedure (WLTP) (Tutuianu et al., 2013, Ciuffo et al., 2015, Commission Regulation (EU) 2017/1151, 2017) the evidence so far suggests that the emissions gap has not completely disappeared (Pavlovic et al., 2018) (ICCT 2021) (Dornoff et al., 2020). In this regard, recent EU regulation (2019/631/EU) introduces monitoring of energy and fuel consumption in real-world driving to curb the discrepancies between real-world and official emissions.

Increasing interest is growing in reducing fuel consumption over real traffic conditions (Mamarikas et al., 2022). Real-world driving involves multiple factors that influence both vehicle operation and drivers’ behavior (Pavlovic et al., 2020) (He et al., 2020b) (Mogno et al., 2020) (Fiori et al., 2019), and a substantial effort is made to model or quantify them through complex tests on the roads (Othman et al., 2019). On the other hand, real-world driving conditions cannot be fully reproduced during type-approval procedures or incorporated into standard chassis-dynamometer laboratory tests due to the inevitable variances in all the influencing parameters defining the side conditions. For instance, driving cycles of fixed velocity profile (also called here speed cycles) are used to establish the official values for the vehicle energy performance, leaving out the role of the driver. The specific acceleration style implicitly assumed during these tests does not necessarily match the whole variety of real-world driving patterns, which in fact can diverge significantly from those expected.

The driving style modulates certain key aspects of the vehicle performance (Barothi et al., 2018). In particular, the longitudinal acceleration has a clear impact on the energy consumption and CO_2_ emissions. The heterogeneity found in real-world acceleration styles could be among the main causes of the CO_2_ emissions gap, and this study will try to elucidate to what extent. This variability is explained both by inter-driver heterogeneity –different drivers show different acceleration patterns-, but also by variations on the behavior of every single driver: The same driver shows different CO_2_ footprints depending both on the vehicle and on a pool of different factors such as weather and traffic conditions, state of the road, driver’s mood and urgency, among others (Fiori et al., 2018).

The scope of the present study is to further investigate and quantify the actual impact of a driver’s acceleration style on real-world CO_2_ emissions and energy consumption. In particular, we study the direct link between the driver’s acceleration aggressiveness and CO_2_ emissions. To facilitate the analysis, we first establish a common reference benchmark for the description, assessment and comparison of different driver behaviors. The WLTP laboratory test procedure, used to evaluate the CO_2_ emissions and fuel consumption of light-duty vehicles in the EU, is optimal for this purpose. The corresponding Worldwide Harmonized Light Vehicle Test Cycle (WLTC) specifies the speed profile that must be followed, within certain tolerances, during the official emissions test, and it can be considered a common framework for all the vehicles in the EU fleet. In this way, we will be able to assess here the impact of the driving aggressiveness exhibited by drivers under real world conditions on the CO_2_ emissions as benchmarked to the WLTC cycle.

Concerning the characterization of the driver’s driving style, our analysis is based on the procedure designed by Makridis et al. (Makridis et al., 2022). In this framework, the driver characterization and the driving style reproduction are achieved through the definition of the Independent Driving Style metric (IDS), separating the driver’s behavior from the vehicle characteristics. The characterization is based exclusively on the analysis of the acceleration pattern, while the gearshifting style is not taken into account. The approach introduces a statistical description of the driving behavior to account for the stochasticity in the acceleration pattern of each driver. Additionally, we introduce here a novel methodology to modify the reference speed cycle (in our case, the WLTC) according to different specific driving styles, carrying out computational simulations to quantify the variations in CO_2_ emissions from different drivers. A clear advantage of our robust methodology is that the quantification of the driving style in terms of CO_2_ emissions is established independently of the vehicle, capturing at the same time the heterogeneity of the driver’s behavior.

The structure of the paper is the following. Section 2 provides a literature review on the driving style characterization and the role of the driver’s acceleration pattern. Section 3 describes the methodology used to characterize the driver’s behavior through the novel IDS metric. First, we assess the acceleration aggressiveness of the WLTC against real world drivers. Then, a new synthetic WLTC-based speed cycle is generated for each specific driver. Finally, we propose a method to quantify the impact of stochasticity and driver’s heterogeneity on the CO_2_ emissions. Section 4 presents the results of our analysis, presenting the CO_2_ emissions variability of different drivers benchmarked against the WLTC reference cycle. In Section 5 we additionally discuss the relationship between acceleration aggressiveness and the amount of CO_2_ emissions. Finally, Section 6 draws general conclusions and thoughts for future work.

## 2 Literature review

The characterization of the driving style has been tackled in the literature through the analysis of different experimentally acquired features (Li et al., 2017) (Van Ly et al., 2013) (Tawfeek and El-Basyouny, 2019) (Zhao et al., 2020) (Yan et al., 2019) (Lee and Jang, 2019) (Feng et al., 2017) (Eboli et al., 2016). A complete review of literature on the topic can be found in (Makridis et al., 2022) and references therein. Liu quantified the speed profile aggressiveness based on the speed fluctuations encountered (Liu et al., 2015). More recently, Martinelli et al. (Martinelli et al., 2020) employed an advanced machine learning procedure to profile the driving style by instantaneous signals from the vehicle. Based on the acceleration of the vehicle, Jurecki and Stanczyk (Jurecki and Stańczyk, 2021) were able to characterize the driving style under various road conditions, obtaining distribution functions that were road specific. Tanvir et al. (Tanvir et al., 2018) employed an ad-hoc metric to analyze the effect of the driving style on the vehicle performance, addressing the heterogeneity both among different drivers and among different vehicles. Barothi et al. (Barothi et al., 2018) explored the influence of the speed profiles gathered from a driver on the vehicle performance. In a similar way, Albayrak Serin and Ozpinar (Albayrak Serin and Ozpinar, 2018) analyzed the effect of the speed over the energy consumption of hybrid electric vehicles. However, most of the studies on this area focus on recognizing or evaluating the driving style from gathered experimental data, rather than simulating the performance of the vehicle under new scenarios determined by different driving behaviors.

The use of theoretical microsimulation models is an appealing solution to reproduce different driving styles based on the elaboration of new trajectories (Laval et al., 2014) (Xu et al., 2015) (Javanmardi et al., 2017). Several works in the past have considered the consequences of the acceleration diversity in both energy and traffic-related studies (Makridis et al., 2020a) (He et al., 2020a) (Makridis et al., 2020b). In particular, Berry (Berry, 2010) modelled the impact of the velocity on the fuel consumption, but the speed profiles used in the simulations were retrieved from a generic database and were not characteristic of any specific driver. Ericsson analysed the relation between 16 independent driving pattern factors related to different driving aspects and the fuel consumption and exhaust emissions (Ericsson, 2001). Addressing the problem of specific driving behaviors is a complex task due to the intricate play among several factors that depend both on the vehicle and on the driver, and which dim the acceleration pattern.

In a recent study, Makridis et al. (Makridis et al., 2022) proposed a model to separate the driver’s behavior from the vehicle’s characteristics. The innovative approach consisted in characterizing each driver’s driving style via a single distribution function of a metric that resemble the acceleration aggressiveness. As a main benefit, this so-called Independent Driving Style metric (IDS) is speed-normalized and vehicle-independent, which allows the characterization of all the positive acceleration events from the driver on a single basis. Indeed, from the comfort point of view, the same acceleration value could be considered as aggressive or conservative depending on the range of velocities where it took place. For instance, an acceleration value of 2 m/s^2^ might seem reasonable when the vehicle moves at 20 km/h, but it would be totally unrealistic at high speeds (e.g., 120 km/h) for the vast majority of conventional vehicles. While the normalization across speed ensures objectiveness for regions of different power capacity, the normalization across vehicles ensures objectiveness for the same driver driving different vehicles.

Despite progress in the driver’s characterization, the simulation of the impact that a specific driving style might have on the vehicle energy performance (and hence CO_2_ emissions) remains an open issue that this study intends to cover. This challenge can only be accomplished with a methodology that allows the modification of the speed profile, creating synthetic trajectories according to the driver’s characteristics. This is a key contribution of the present work, which will in addition lead to the assessment of the acceleration aggressiveness assumed during the WLTC cycle in comparison to drivers in real-world conditions.

## 3 Methodology

To facilitate a better understanding of the present analysis, we provide a set of definitions of the most frequently referred concepts.

• **Driving cycle**: A predefined speed trace over time or distance that represents a complete trip and which is repeated in different experiments.
• **Speed profile**: A series of speed values as function of travelled distance or trip time, obtained from an observed or simulated vehicle trajectory.
• **Vehicle trajectory**: The connection of all positions and corresponding times where the vehicle has been during the course of a trip.
• **Driving style**: A distribution of IDS-metric values that characterizes the acceleration aggressiveness of the driver.
• **IDS metric**: Independent Driving Style metric, reproduces the aggressiveness of free-flow acceleration events independently of the vehicle and the speed range.
• **Free-flow acceleration**: Acceleration that takes place when the driver does not drive behind any other vehicle and can accelerate at his/her will.

During this study, we consider the WLTC as our reference driving cycle. The vehicle performance regarding level of pollutants, CO_2_ emissions and fuel energy consumption are generally assessed on laboratory tests. In view of the disparity of tests around the world, the United Nations Econonmic Commission for Europe (UNECE) initiated in 2009 the development of a worldwide harmonized light-duty standard, which resulted in 2017 in the World harmonized Light-duty vehicle Test Procedure (WLTP) (Tutuianu et al., 2015, Ciuffo et al., 2015, Commission Regulation (EU) 2017/1151, 2017). This test substituted the depreciated European Type-Approval procedure (NEDC), which had proven to provide official values far from the real world existing data (Ntziachristos et al., 2016). The importance of realistic driving cycles in emissions and fuel consumption measurements had been recognized very early, and projects like ARTEMIS created representative driving profiles for the European traffic situations (André, 2004). In particular, the new driving cycle adopted in WLTP (the WLTC cycle) has proven to better represent realistic driving conditions than the previous NEDC one.

**Fig. 1:**
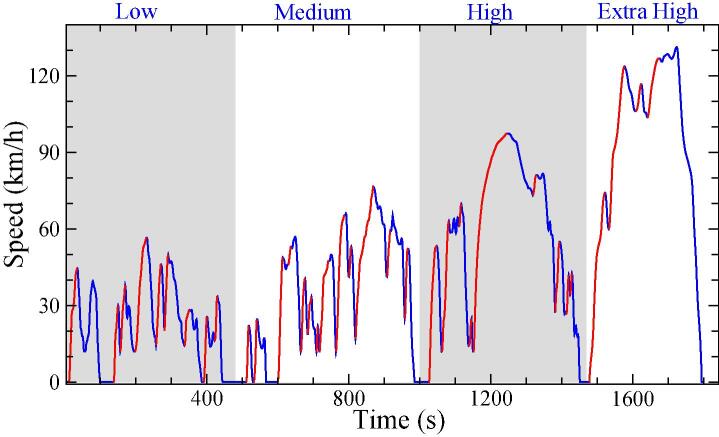
Worldwide Harmonized Light Vehicles Test Cycle (WLTC) speed profile as a function of time for Class 3b. Acceleration parts are displayed in red color. (For interpretation of the references to color in this figure legend, the reader is referred to the web version of this article.)

The WLTC cycle is based on a specific speed profile in the time domain, as represented in Fig. 1. In the present paper we define a new hypothetical reference driver (WLTC-driver) from the WLTC speed profile. At the same time, we consider the driving data retrieved from a sample of 20 drivers in real-world conditions during the experimental campaign carried out at the Joint Research Center (JRC). Based on the driving style analysis carried out by Makridis et al.(Makridis et al., 2022), we modify the reference cycle creating new speed profiles adapted to the characteristics of each of the 20 drivers. In practice, this means different CO_2_ emissions estimates for each driver.

**Fig. 2:**
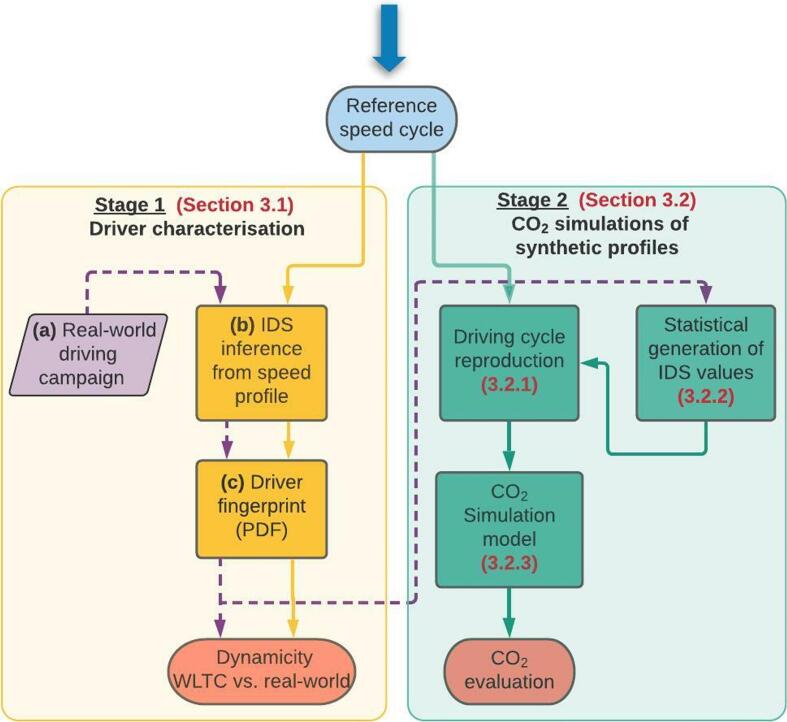
Flowchart of the procedure followed in this study for assessing the characterization of WTLC’s driving style and the impact on CO_2_ emissions. Labels in brackets refer to the sections/subsections where such a task is described.

Fig. 2 summarizes the steps of the proposed methodology, which can be grouped into two stages. In the first one, we analyze the acceleration aggressiveness of the drivers in real world conditions as compared to the driving style defined in WLTC. The starting point is the WLTC speed cycle, where each positive acceleration event is analyzed regarding the acceleration aggressiveness by means of an ad-hoc defined metric. The ensemble of metric values gathered from the speed profile can be considered as the fingerprint of the driving style assumed, and can be used for benchmarking the dinamicity of the 20 drivers in the experimental campaign.

In the second stage, a new speed cycle is created for each individual based on his/her real-world driving performance. The performance of the vehicle throughout this cycle yields the CO_2_ emission pattern that can be associated to each driver. The methods employed for the simulations and for the synthetic elaboration of the new overall trajectories will be described in the following sections.

### 3.1 Driver characterization

In general, driving is simulated under two prevalent conditions, free-flow and car-following (Ambros and Kyselý, 2016). Free-flow assumes that the vehicle is moving freely without any obstacle ahead (another vehicle, traffic light etc.), while car-following implies that the vehicle is following another vehicle ahead. According to Makridis et al. (Makridis et al., 2022), it is during the acceleration under free-flow conditions when the driver shows a more distinctive driving behavior. Observing a driver for a long period, one can identify many such events.

Makridis et al. (Makridis et al., 2022) introduced a new metric for the characterization of the aggressiveness of each free-flow acceleration event, called the Independent Driving Style (IDS). Practically, the IDS formulation describes the aggressiveness of the acceleration demanded by the driver, and is achieved by employing a two-fold normalization procedure. In a first stage, the instantaneous acceleration measured is normalized to the vehicle’s power capability, rendering the magnitude that Makridis et al. call *ds*:

(1) $$\mathrm{ds}\left(t;v\left(t\right)\right)=\frac{a(t)}{a_{\mathrm{cp}}\left(v\left(t\right)\right)}$$

The *a_cp_(v(t))* reference value, the acceleration potential, is a function of the instantaneous speed, and is constant for all drivers on the vehicle and at the same time different for each vehicle. The inter-driver and intra-driver variability in terms of acceleration aggressiveness leads to a variable range of normalized acceleration values (*ds*). As demonstrated in (Makridis et al., 2022), this range of variability still depends on the average speed where the acceleration event takes place. Indeed, as mentioned in the introduction, a similar acceleration could be characterized differently depending on how fast the vehicle was moving. This residual correlation is eliminated by means of a second normalization procedure based on the maximum (*f_max_*) and minimum (*f_min_*) normalized (averaged) acceleration values found for all the acceleration events from all drivers at a given average speed, rendering the IDS metric:

(2) $$\mathrm{IDS}=\frac{{\overset{¯}{\mathrm{ds}}}_{\mathrm{norm}}\left(\overset{¯}{v}\right)-f_{\min}\left(\overset{¯}{v}\right)}{f_{\max}\left(\overset{¯}{v}\right)-f_{\min}\left(\overset{¯}{v}\right)}$$

In this way, IDS is a speed-normalized and vehicle independent metric that serves at the same time as a reference for the characterization of the aggressiveness of each acceleration event, irrespective of the speed where it took place, and as a basis for the direct comparison of the driving behavior (acceleration style) between different drivers.

Using a local mininum-maximum algorithm to detect the acceleration events, the corresponding IDS values are computed and collected. In view of the heterogeneity in the acceleration aggressiveness that each driver shows in real world, we characterize each driver’s acceleration style by a distribution of IDS values. This distribution is considered as the driver’s fingerprint, and, as presented later, it is used to simulate the driver’s behavior.

**Fig. 3:**
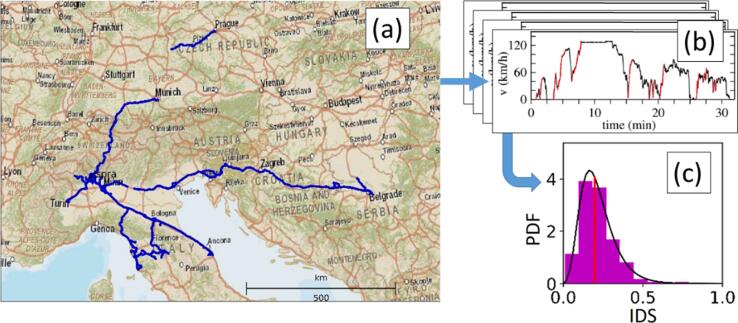
Summary of the drivers' characterization in Makridis et al *(*Makridis et al., 2022*)*. (a) Part of the trips in the experimental campaign are collected. (b) The corresponding speed profiles for a certain driver are analyzed regarding the acceleration style, assigning a single IDS value to each free-flow acceleration event (in red color). (c) All the IDS values collected from a certain driver are used to create the characteristic IDS distribution that defines each driver’s behavior. For more details, the reader is referred to the original publication. (For interpretation of the references to color in this figure legend, the reader is referred to the web version of this article.)

Fig. 3 illustrates an example of the procedure followed to characterize a given driver. The (a), (b) and (c) panels correspond to the (a), (b) and (c) boxes in Stage 1 of Fig. 2. In a first instance (a), the data of the driving campaign (or alternatively the WLTC cycle) are collected. In (b), the resulting speed profiles are processed, analyzing the positive acceleration events. For each of these events, the IDS value is calculated. Finally (c), gathering the IDS values from all the free-flow acceleration events in the speed profile, we obtain a fingerprint of the driver’s style in the form of a Probability density function (PDF). A more detailed description of the IDS formulation is provided in (Makridis et al., 2022).

Given the heterogeneity of the driver’s driving style, the assessment of the aggressiveness in the acceleration among different drivers is not straightforward. Therefore, we define a new *R*-index that incorporates stochasticity for the characterization of the aggressiveness of each driver. This index represents a ranking for the number of times that a specific driver accelerates faster than other drivers, with high *R*-index values associated to more aggressive accelerations. This comparative procedure is performed on a one-to-one basis and must be repeated a large amount of instances (10^6^) to generate a representative sample.

(3) $$R_{i}=\frac{1}{N_{c}}\sum_{j\neq i}^{21}\sum_{n}^{N_{c}}u\left({\mathrm{IDS}}_{i}-{\mathrm{IDS}}_{j}\right)$$

Here, *u(x)* represents the Heaviside function, while each *IDS_m_* value is randomly generated using a Monte Carlo statistical method to solve the inverse problem of a PDF log-norm function. The *R*-index presents limiting cases at *R_i_* = 0 (the driver always accelerates softer than the rest of the drivers) and *R_i_* = 20 (the driver is always faster than the others). Therefore, a value >10 indicates that the driver is in the top 50% regarding acceleration aggressiveness.

### 3.2 CO_2_ simulations of synthetic profiles

The procedure for evaluating the CO_2_ emissions along a driver-adapted WLTC cycle consists of three main steps that are already indicated in Fig. 2. First, we produce a new driving cycle (section 3.2.1), according to some IDS values that are generated statistically (section 3.2.2). We later quantify the CO_2_ emissions based on the simulations from the CO_2_MPAS model (section 3.2.3).

#### 3.2.1 Driving cycle reproduction

**Fig. 4:**
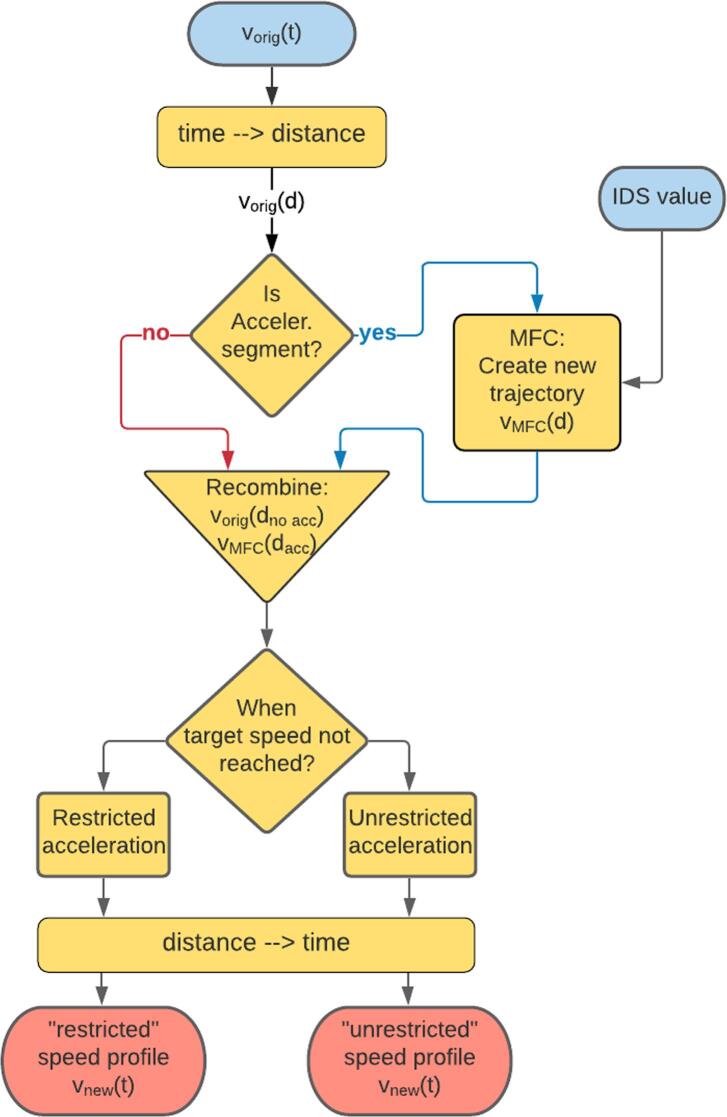
Flowchart for the modification of the speed profile according to the IDS value.

The aim of driving cycle reproduction is to create a synthetic, yet realistic individual WLTC speed profile for each driver by modifying the acceleration events according to the driver’s individual IDS profile. The proposed methodology labelled 3.2.1 in Fig. 2, is summarized in Fig. 4.

**Fig. 5:**
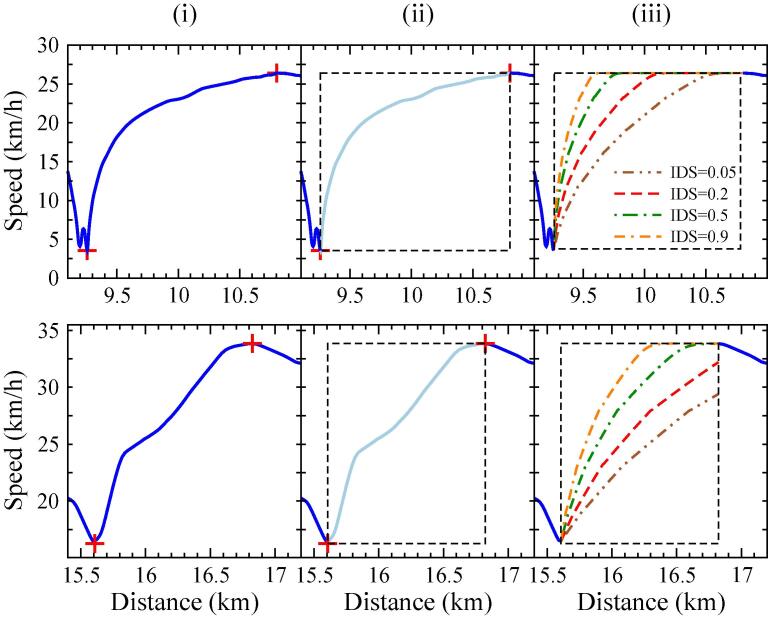
Illustration of how the new MFC trajectories are generated from four different IDS values (0.05, 0.2, 0.5 and 0.9) during a soft (upper panels) and a strong (lower panels) acceleration event. Panels (i), (ii) and (iii) represent the three steps followed for the MFC-generated trajectories: (i) We identify the location of the minima and maxima of the speed profile, which correspond to the initial and final points of each trajectory. (ii) The minima and maxima determine the initial speed, desired (or target) speed, initial position and final position. The WLTC profile is eliminated, creating a gap in the speed profile. (iii) We generate the new MFC-trajectories using a fix specific IDS value. Depending on the aggressiveness of the original acceleration event and on the IDS value, the target speed might be reached (upper panels) or not (lower panels for IDS > 0.2) at the end of the event. Background rectangles are defined by *d_i_*, *d_f_*, *v_i_* and *v_t_*, as explained in the text.

In order to fix the total distance of the cycle, the speed is translated from time to distance representation. Then, we generate new pieces of the speed profile that will substitute the original parts. This is accomplished in the three steps illustrated in Fig. 5:

(i) First, we detect the acceleration events on the reference WLTC that correspond to free-flow conditions. From these acceleration events, we calculate the IDS values that will serve to construct the characteristic IDS distribution for each driver.
(ii) Then we remove these parts, creating gaps on the distance profile of the WLTC cycle.
(iii) Finally, we use a vehicle dynamics-based model to reconstruct the gaps according to the IDS distribution of the driver. The MFC model (Makridis et al., 2019) (He et al., 2020b) is used for the simulation of these partial trajectories. Each acceleration event (gap) has a specific length, determined by the original acceleration in the WLTC. The new acceleration trajectories are generated from a driving style parameter related to the IDS metric (Makridis et al., 2022). Hence, each value of the IDS metric determines a different acceleration profile.

The two examples shown in Fig. 5 correspond to a smooth (top panel) and an aggressive (bottom) acceleration event.

**Fig. 6:**
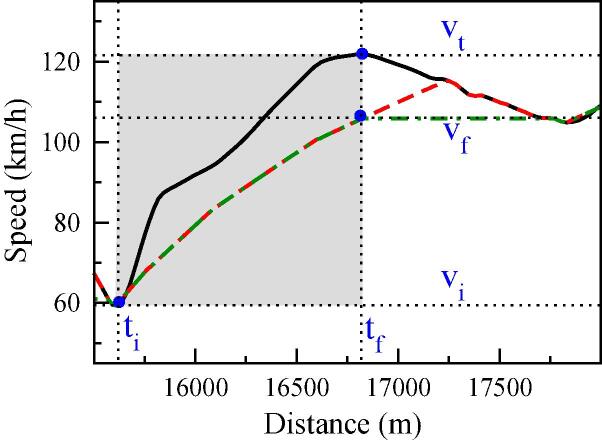
Speed profiles for acceleration event 34, showing definition of parameters *d_i_*, *d_f_*, *v_i_*, *v_t_* and *v_f_*. WLTP (solid black), restricted (dashed-dotted green line) and unrestricted (dashed red line) speed profiles. (For interpretation of the references to color in this figure legend, the reader is referred to the web version of this article.)

Each MFC-generated trajectory on the right-hand panel represents a different acceleration aggressiveness, characterized by a different IDS value of 0.05, 0.2, 0.5 and 0.9. From the reference WLTC profile we define the initial (*d_i_*) and final distances (*d_f_*), -initial and final times (*t_i_* and *t_f_*)-, and initial (*v_i_*) and target speed (*v_t_*) of each acceleration event. Depending on the aggressiveness of the driver, the simulated profiles reach the desired speed in distances very likely different from the reference (WLTC) one. The upper panel describes a quite smooth acceleration on the original WLTC, and the four generated profiles reach the target speed before the end of the event. Meanwhile, the lower panels show a more aggressive acceleration in WLTC, and this time some of the IDS values (i.e., IDS = 0.05 and IDS = 0.2) yield trajectories where the vehicle has not reached yet the target speed at the end of the event. This would yield discontinuities when merging the modified speed profile with the original one. Two different strategies, displayed in Fig. 6, are proposed to circumvent this limitation:

##### 3.2.1.1 Restricted acceleration

The first strategy consists of maintaining the final speed outside the acceleration event until we encounter again the original speed profile. The acceleration is in this case limited to the region defined between *d_i_* and *d_f_* distances, and this strategy will be referred to as “restricted” throughout the study. In real driving conditions, this enforcement may represent an obstacle in the road (such as traffic lights, curves, etc.) that forces the driver to stop the acceleration at the final distance.

##### 3.2.1.2 Unrestricted acceleration

In the “unrestricted” strategy, the driver keeps accelerating beyond the final distance *d_f_* until reaching again the original speed profile. In this strategy, we ignore any possible hindrance for reaching the desired target speed. A typical example of this would be an acceleration event while driving on a highway, were the road is typically free of any other limitation than the speed limit. This type of acceleration strategy leads smaller differences from the original speed profile as compared to the restricted acceleration strategy.

#### 3.2.2 Statistical generation of IDS values

The next question concerns the selection of the IDS values used to generate a speed profile that reproduces the desired driving style. This step is also shown in Fig. 2, and connects the fingerprints (PDF functions) of the drivers in the experimental campaign with the driving cycle reproduction block (3.2.1). We employ the following two approaches:

1) Deterministic-IDS approach: This approach, that will be adopted in section 4.2.1, is intended for exploring the impact of possible driving styles (defined by a single IDS value) on the CO_2_ emissions. It consists of keeping the IDS value fixed throughout the complete WLTC cycle. The main advantage is the direct correlation established between the IDS metric and the CO_2_ emissions value. This illustrates the impact of the acceleration aggressiveness on the greenhouse emissions. In this sense, we could think of the CO_2_ emissions as an additional metric that could be used for categorizing the driving style, with a direct correspondence to experimental observations. At the same time, this approach presents a clear limitation in taking a constant IDS value, given the heterogeneity found in real-world drivers.

2) Probabilistic approach: In the case of drivers under real conditions, a statistical approach seems more adequate for describing the stochasticity of the human driving behavior. The IDS values are generated from the inverse cumulative distribution function of a lognormal PDF, which can be computationally implemented using Monte Carlo methods. The strategy uses the same parameters that were used to model the driving style. The disadvantage of this approach is that it involves a higher complexity in the modelling, and yields in most cases, cumbersome simulations due to the large number of statistical repetitions required. To overcome this limitation, one can combine the (parametrized) IDS/CO_2_ correlation of the deterministic approach with the probabilistic functions for the IDS characterization of the driving style, as will be shown in section 4.2.2.1. This method is not as accurate as the genuine probabilistic method (4.2.2.2), but reduces considerably the computational load.

#### 3.2.3 CO_2_ simulation model

The proposed methodology requires a validated tool that simulates the vehicle’s behavior and quantifies consequently the amount of emissions, as pointed out in Fig. 2. Here we employ the CO_2_MPAS package (Ciuffo and Fontaras, 2017) (Tsiakmakis et al., 2017) (Fontaras et al., 2018). CO_2_MPAS is a detailed vehicle simulation model that reproduces the performance of the vehicle under different operational conditions, predicting fuel consumption and CO_2_ emission values for any desired input speed profile. CO_2_MPAS incorportates a default gearshifting prediction model that has been used for all the drivers during the generation of the new acceleration trajectories. The CO_2_MPAS simulation tool has demonstrated its accuracy in the prediction of CO_2_ emissions under different official cycle tests (WLTP, NEDC), with low errors of the order of < 2% and standard deviation ∼ 5% for the NEDC cycle. Concerning real-world trips, and despite the limited quality of input data in such campaigns, CO_2_MPAS has also demonstrated an outstanding prediction capability, with overall biases below 3% and uncertainties in the range of 5–7% (Mogno et al., 2020) (Makridis et al., 2020c).

### 3.3 Assessment strategy

After introducing the basic concepts of the driving style that will be used in this study, we can summarize the procedure followed to evaluate the impact of driver’s heterogeneity on CO_2_ emissions benchmarked to the WLTC cycle:

• In a first step we analyze the WLTC speed profile as measured in the laboratory, constructing the corresponding IDS distribution from the free-flow acceleration events found therein. We consider that such distribution belongs to a virtual driver, i.e., the WLTC-driver, and contrast it against drivers’ distributions from real world. This will give us an idea of the variability in acceleration aggressiveness that can be found in real world referenced to the WLTC pattern.
• Secondly, we evaluate the CO_2_ emissions that stem from assuming a constant IDS value throughout the WLTC-based speed cycle. This fixed-IDS approach does not represent the heterogeneity of a real driver, but serves as a way to establish a first correlation between IDS and CO_2_ emissions.
• In parallel, using Monte Carlo generating methods, we create new synthetic (WLTC-based) speed profiles that are adapted to each of the 20 drivers.
• Finally, we model the vehicle’s energy consumption over each of these synthetic speed profiles and evaluate the corresponding CO_2_ emissions. We then obtain 20 different probabilistic CO_2_ emissions distributions (one per driver), which are finally compared with the WLTC reference value.

## 4 Results and discussion

### 4.1 WLTC-driver

This first block presents the results of the characterization of the WLTC for the Class 3B vehicle used in this work. The complete cycle (see Fig. 1) lasts 1800 s for a total distance of 23.27 km, with a dynamic speed profile based on continuous accelerations and decelerations. Attending to the minimum duration and minimum speed increase, we identify a total of 31 free-flow acceleration events that are detailed in Table 1, calculating in each case the corresponding IDS metric.

**Table 1:** Median IDS ($\hat{\text{IDS}})$, median speed ($\hat{v}$), initial time (*t_init_*) and duration (Δt) of each free-flow acceleration event in WLTC.

Event	$\hat{\text{IDS}}$	$\hat{v}$	*t_init_*	Δt	Event	$\hat{\text{IDS}}$	$\hat{v}$	*t_init_*	Δt
*#*		*[km/h]*	*[s]*	*[s]*	*#*		*[km/h]*	*[s]*	*[s]*
*1*	0.158	30.8	15	18	17	0.081	40.2	721	27
*2*	0.086	31.2	61	16	18	0.213	52.5	767	18
*3*	0.208	20.4	140	9	19	0.329	48.4	802	4
*4*	0.121	28.6	158	11	20	0.053	53.9	824	40
*5*	0.088	41.6	202	25	21	0.147	57.6	910	13
*6*	0.206	32.5	259	10	22	0.557	40.4	959	6
*7*	0.292	40.7	283	8	23	0.270	36.3	1028	15
*8*	0.017	24.5	339	10	24	0.227	44.5	1066	28
*9*	0.300	10.3	392	7	25	0.218	23.5	1143	3
*10*	0.292	20.9	417	11	26	0.031	74.9	1158	47
*11*	0.362	9.2	512	5	27	0.349	39.4	1384	8
*12*	0.256	8.2	534	6	28	0.280	39.5	1416	13
*13*	0.362	28.0	602	11	29	0.131	51.7	1486	31
*14*	0.176	32.0	667	8	30	0.287	95.3	1538	33
*15*	0.104	30.3	686	9	31	0.047	117.6	1646	20
*16*	0.157	19.1	710	3					

**Fig. 7:**
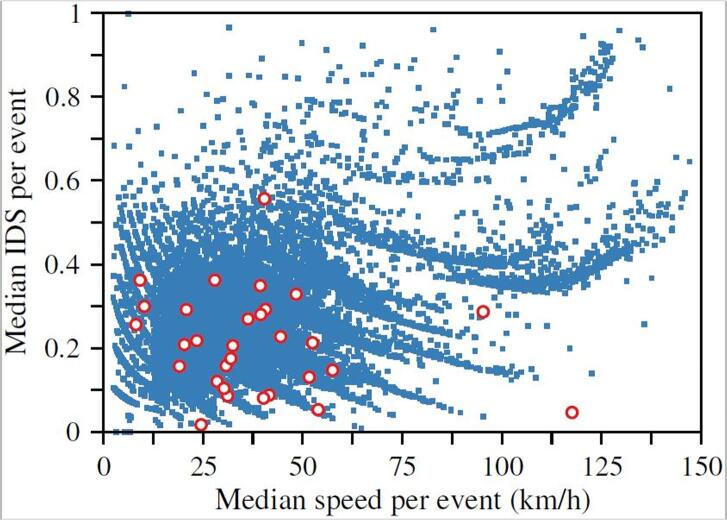
Independent Driving style (IDS) parameter obtained for each acceleration event as a function of the median speed for the WLTP speed profile. Red circles denote WLTC events, while blue points correspond to the free-flow acceleration. (For interpretation of the references to color in this figure legend, the reader is referred to the web version of this article.)

Fig. 1 in section 2 highlights the acceleration events that fulfill the requirements of free-flow conditions as defined in Section 2.1.1 of (Makridis et al., 2022). The IDS values of these acceleration events present a clear stochastic character. These values are plotted in Fig. 7 together with the data obtained from the 20 drivers in the driving campaign at the JRC-Ispra. The points representing the WLTC events are scattered in the region most commonly explored by the real-driving events. Nevertheless, the WLTC values are clearly constrained to the region below IDS = 0.55, as can be also confirmed in Table 1. This suggests a possibly conservative acceleration style in the WLTC driving characterization, as a consequence of the restricted duration of the cycle (30 min), and therefore the limited number of acceleration events incorporated.

**Fig. 8:**
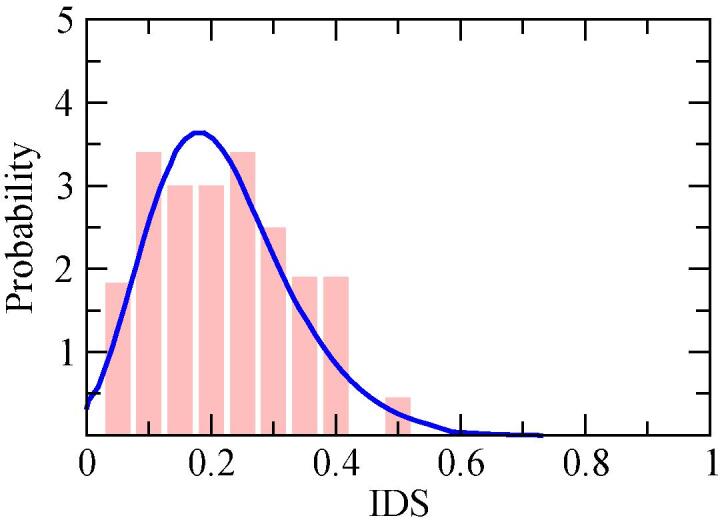
Histogram and fitted lognormal Probability Distribution Function (blue line) along IDS metric for the WLTC-driver, with parameters *loc* = -0.261, *scale* = 0.467, *s* = 0.241 and a median value $\hat{\text{IDS}}=0.198$*.* (For interpretation of the references to color in this figure legend, the reader is referred to the web version of this article.)

This inhibition is better visualized in the histogram of the IDS values gathered from the WTLC cycle, Fig. 8. Following the parametrization detailed in (Makridis et al., 2022), we obtain a Probability Distribution Functions (PDF) that has a lognormal form, with a median value $\hat{\text{IDS}}=0.198$. Unfortunately, the restricted number of 31 events represents a poor statistical set in contrast to the thousands of events recorded from the drivers in the experimental campaign, which leads to the cut-off at IDS = 0.5 in the corresponding PDF function.

**Fig. 9:**
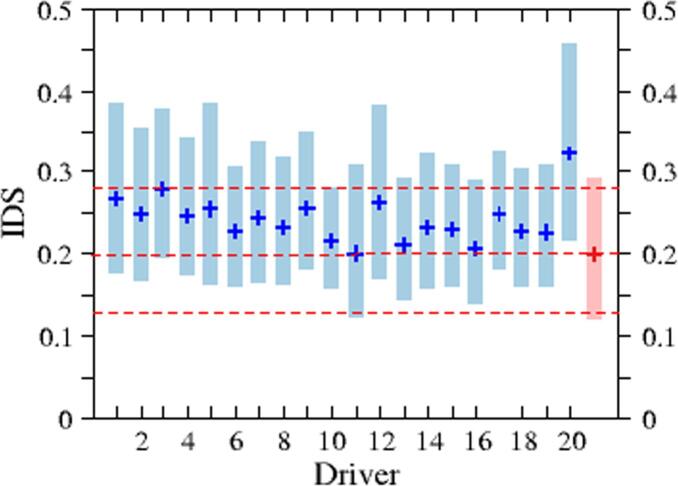
Bars show the 25% and 75% quartiles of the IDS statistical distribution for the 20 drivers from the experimental campaign (blue color) and the WLTC cycle (red color). Symbols indicate the median value. Red horizontal dashed lines included as a reference of the WLTC for better comparison with individual drivers. (For interpretation of the references to color in this figure legend, the reader is referred to the web version of this article.)

Fig. 9 presents further insights in the comparison between WLTC and the 20 drivers. The figure displays the quartiles of the statistical distribution of the IDS for each driver, including the WLTC. The bar covers the region [25%-75%], while the symbols are included to represent the median of the distribution. We can conclude that the WLTC cycle is clearly more conservative than the 20 drivers in the sample. The extension between the 1st and 3rd quartiles in WLTC is similar to the rest of the drivers, which at the same time indicates the robustness of the original sampling used to build the WLTC cycle.

**Fig. 10:**
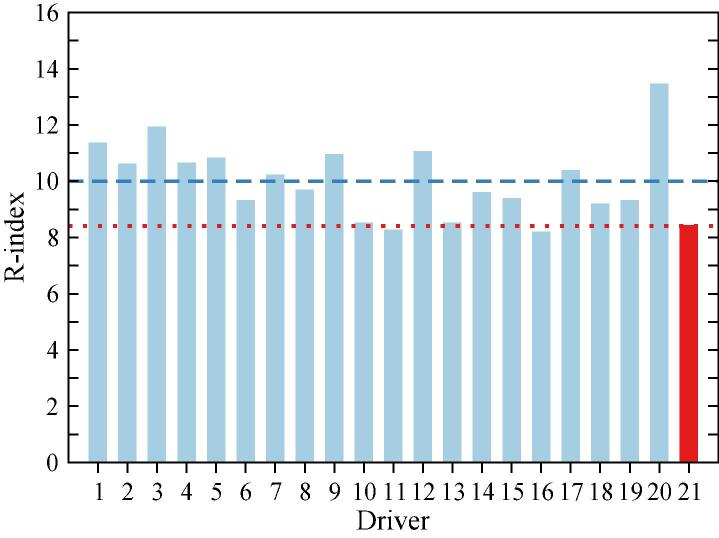
Comparison of the *R*-index that characterises the driving style for each driver in the experimental campaign, including the WLTC as the 21st case. Blue dashed line represents average *R* value for the 20 drivers in the sample. Red dots indicate WLTC *R*-index. (For interpretation of the references to color in this figure legend, the reader is referred to the web version of this article.)

Fig. 10 presents a ranking of driving styles according to the aggressiveness based on the new metric *R*-index, that was already introduced in section 3.1. Again here, the WLTC-driver presents a lower *R*-index than most of the 20 drivers in the sample, with the only exception of D11 and D16. In any case, the WLTC *R*-index value is well below the average (*R = 10*) that divides the aggressive drivers from the conservative ones. This means that the driving style forecasted by the WLTC shows most of the times a timider acceleration behavior than what one would expect from the real-driver’s style. This is most likely a consequence of the mentioned cut-off that the WLTC-based data sets present.

In view of the Fig. 7, Fig. 9, Fig. 10, one can conclude that the driving style of the WLTC is consistently timider than the one found from the 2018 JRC sample. However, it is difficult to go further in the quantitative analysis of the different driving styles, since the probability distribution functions that characterise each style are not accurate enough due to the few number of free-flow acceleration events in the test cycle.

### 4.2 Evaluation of energy consumption and CO_2_ emissions

The fingerprint of each driver is a distribution of IDS values. Some values indicate more dynamic acceleration than others, and this could be expected if we think that each individual might behave in a different manner -from the acceleration point of view- given the driving circumstances. Statistically, this is reflected in his/her IDS distribution, with a probability density function (PDF) that resembles a rather broad lognormal distribution. Given this stochastic nature of the IDS metric, evaluating the impact on CO_2_ emissions that each acceleration style has is all but simple. Here we employ the two different methods introduced in section 3.2.2.

#### 4.2.1 Deterministic-IDS approach

**Fig. 11:**
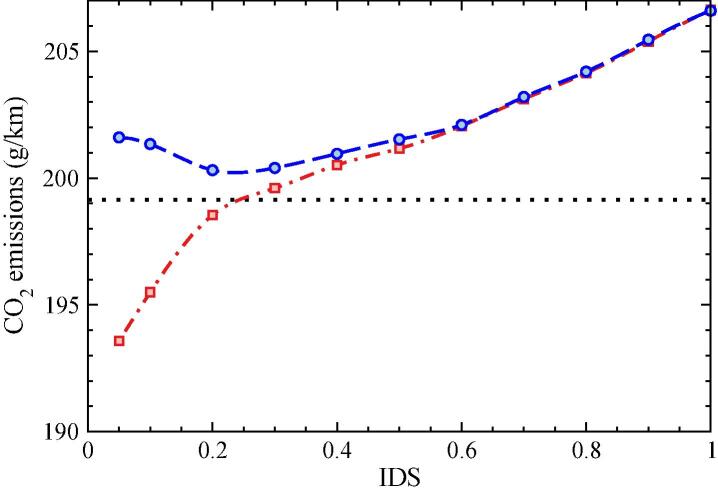
CO_2_ emission values as a function of the fixed-IDS parameter for the restricted (dashed-dotted red line, open squares) and unrestricted (dashed blue line, bullets) strategies. The WLTC speed profile is modified according to the specific IDS value. Type-Approval CO_2_ emissions for the reference WLTC cycle is also displayed in horizontal dotted line. (For interpretation of the references to color in this figure legend, the reader is referred to the web version of this article.)

The impact range of all possible driving styles on the CO_2_ emissions is illustrated in Fig. 11. This deterministic IDS-approach consists in fixing a constant IDS value for all the acceleration events in the speed cycle. Repeating the procedure for different IDS values, we can establish a direct correlation between the IDS metric and the average CO_2_ emissions. In this way, we set the limits in the range of emissions that results from keeping a specific driving style for the whole cycle.

The two sets displayed in Fig. 11 correspond to the restricted and unrestricted strategies explained in sections 3.2.1.1 and 3.2.1.2 for merging the new trajectories with the original profile. The first conclusion is that the amount of CO_2_ emissions increases with the aggressiveness of the driving style. For IDS > 0.2, both strategies yield almost identical values, following a quasi-linear dependency and reaching the maximum value of 206.7 g/km at IDS = 1. The mean IDS value of the WLTC (IDS = 0.2) is a break-even point, below which each strategy results in a different speed profile and thus yields a different emissions pattern. In the restricted strategy the energy consumption and CO_2_ emissions have a positive relation to IDS, due to the decline in the speed profile during the passages that follow incomplete acceleration events. On the other hand, the relationship is negative for the unrestricted acceleration strategy, with a global minimum close to IDS = 0.2 that interestingly coincides with the most frequently rated IDS value in the original WLTC.

#### 4.2.2 Probabilistic-IDS approach

Drivers show in real-world conditions a manifold of different driving styles. Statistical lognormal functions are suitable for the description of the IDS metric of each driver. Consequently, the evaluation of the CO_2_ must also be carried out under this probabilistic approach. In practice, this means that instead of using a single IDS value throughout the whole WLTC, as in Section 4.2.1, we should sample the IDS values from the lognormal distribution that characterizes each driver. Following this random generation procedure, we derive the new driver-adapted combined cycle and use CO_2_MPAS to quantify energy consumption and CO_2_ emissions.

Attending to the random generation of IDS values, two different approaches have been adopted. In both cases, the scarcity of free-flow acceleration events in the WLTC (31 events) would negatively impact the statistics. To overcome this limitation we concatenate multiple cycles, creating a long representative one.

##### 4.2.2.1 Direct sampling from fixed IDS results

In this approach we combine the relationship between fixed-IDS and CO_2_ emissions shown in the previous section (see Fig. 11) with the distribution representation (PDF) that defines each driver’s driving style. In other words, we create a long speed profile concatenating fixed-IDS segments, and the number of segments for each IDS value is given by the probability distribution of each driver. From a mathematical perspective, this procedure is equivalent to a change of variable in the driver characteristic PDF function, moving from the original IDS variable to the CO_2_ emissions one. The transformation can only be achieved provided the correlation between IDS and CO_2_, shown in section 4.2.1, is parametrized. The main drawback of this method is that the duration of each segment can be significantly different from the 1800 s duration of the WLTC reference cycle.

**Fig. 12:**
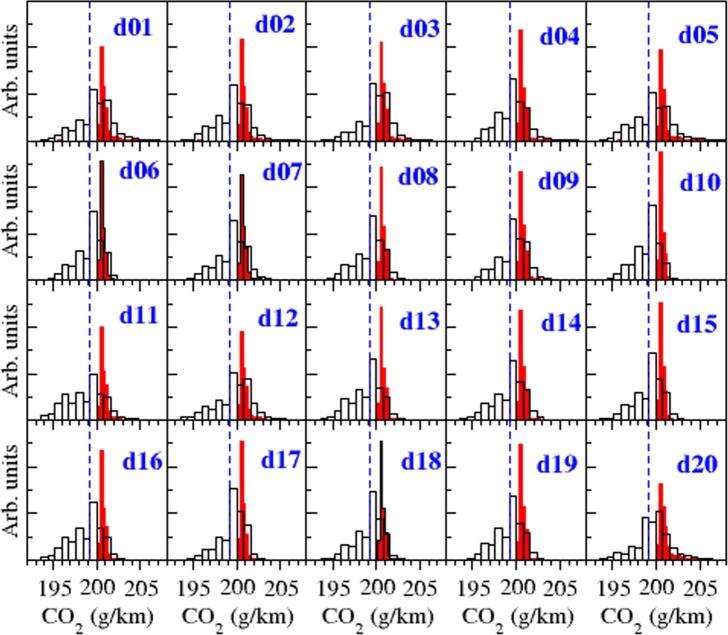
Histograms representing the CO_2_ emissions of the 20 drivers as explained in 3.2.2.1 for the restricted (black empty bars) and unrestricted (red filled bars) acceleration strategies. Dashed blue vertical lines represent CO_2_ emissions in WLTP test (i.e., Type-Approval value). (For interpretation of the references to color in this figure legend, the reader is referred to the web version of this article.)

We have characterized the CO_2_ emissions of the 20 drivers on a statistical approach, obtaining for each driver a CO_2_ emissions histogram. Fig. 12 shows the results for both the restricted and unrestricted acceleration strategies (see 3.2.1.1 and 3.2.1.2). We provide further information in Table 2, which include the average value and the standard deviation of the CO_2_ emission distributions. For clarity, include the median value of each driver’s IDS distribution as found by Makridis et al (Makridis et al., 2022). At first glance, the differences between the average emissions of the 20 drivers are relatively small, compared to the variation of CO_2_ over fixed-IDS values (Fig. 11). The reason behind this lower impact is that most of the drivers present a similar behavior that mainly explores the lower part of the IDS range, and very seldom the highest range (above IDS = 0.5). Despite of it, we find a trend in the results, with Driver 20 –who proved to be the most aggressive driver– presenting the highest CO_2_ emissions value. On the other side, Driver 11 is quite conservative according to Fig. 9, Fig. 10, and casts the lowest emissions in the restricted strategy but considerably high ones for the unrestricted case. This driver is very often accelerating with low IDS values, where both strategies yield opposite trends.

**Table 2:** Average (<CO_2_ > ) and standard deviation (*σ*) values for CO_2_ emissions for the 20 drivers in the test campaign adopting the restricted and unrestricted acceleration strategy. Median values of the IDS distribution for each driver are specified in the second column, while for WTLC $\hat{\text{IDS}}=0.198$.

		**Restricted strategy**		**Unrestricted strategy**	
**Driver**	$\hat{\text{IDS}}$	<CO_2_ > [g/km]	*σ* [g/km]	<CO_2_ > [g/km]	*σ* [g/km]
1	0.266	199.01	1.42	200.97	1.30
2	0.247	198.77	1.32	200.96	1.19
3	0.277	199.13	1.15	200.87	0.96
4	0.244	198.86	1.14	200.82	0.59
5	0.253	198.84	1.57	201.06	1.56
6	0.226	198.40	1.09	200.76	1.17
7	0.242	198.66	1.24	200.86	1.23
8	0.231	198.49	1.14	200.80	1.21
9	0.255	198.88	1.12	200.82	0.87
10	0.214	198.22	1.01	200.72	1.04
11	0.199	197.96	1.62	201.11	2.36
12	0.262	198.86	1.53	201.04	1.68
13	0.210	198.15	1.22	200.83	1.50
14	0.231	198.45	1.25	200.85	1.40
15	0.228	198.40	1.14	200.78	1.24
16	0.205	198.02	1.30	200.87	1.70
17	0.247	198.71	0.98	200.73	0.87
18	0.226	198.37	1.12	200.76	1.24
19	0.225	198.44	1.13	200.78	0.95
20	0.321	199.65	1.46	201.15	1.25

##### 4.2.2.2 Monte Carlo IDS sampling validation

The previous probabilistic approach is based on the assumption that the IDS value is maintained during the 23.27 km of length of each segment (the length of the reference WTLC). To ascertain the validity of this assumption, we include here a second approach, more accurate but also more cumbersome due to the massive number of simulations involved, where we take a different IDS value for each of the acceleration events inside each segment. The method involves the combination of *N_c_* different segments, and the IDS is randomly generated for each acceleration event inside the segment. We use the Monte Carlo method to solve the inverse transformation function problem of a lognormal distribution (Abramowitz and Stegun, 1972, Rubinstein and Kroese, 2017). To assure the convergence in <0.01%, we build the total combined cycle concatenating *N_c_* = 1000 segments.

**Fig. 13:**
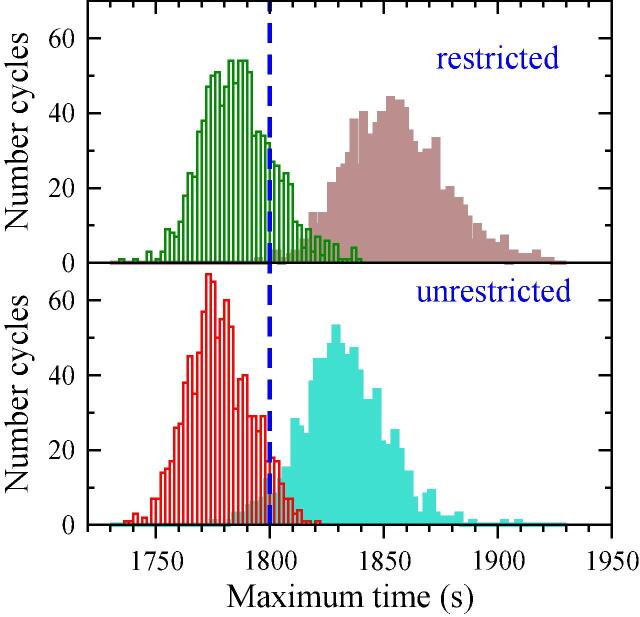
Histograms representing the duration of the segments in the Monte Carlo sampling procedure for the restricted (up) and unrestricted (down) strategies. Empty boxes: Driver 11, Full boxes: Driver 20. Vertical dashed-blue line indicates original WLTC duration. (For interpretation of the references to color in this figure legend, the reader is referred to the web version of this article.)

For the sake of conciseness, we focus on the most aggressive (Driver 20) and the least aggressive drivers. The length of the segments that conform the total cycle is always the same, but since the generated speed profile is different, we also expect a variable duration. Since smaller/larger IDS values will yield longer/shorter segments, respectively, deviations from the standard 1800 s of the WLTC are a good hint of the aggressiveness of the driving style. The total time registered for each segment is plotted in the histogram of Fig. 13, both for the restricted and unrestricted strategies and for drivers 11 and 20. As expected, driver 20 is clearly showing a more aggressive acceleration style, with an average value of 1786 s for the restricted and 1778 s for the unrestricted acceleration, both lower than the WLTC reference time of 1800 s. Driver 11 is in contrast consistently delayed, (1855 s and 1832 s for the restricted and unrestricted schemes). The difference between both strategies is more significant for driver 11 since involves lower values in the IDS distribution, where both strategies diverge.

**Fig. 14:**
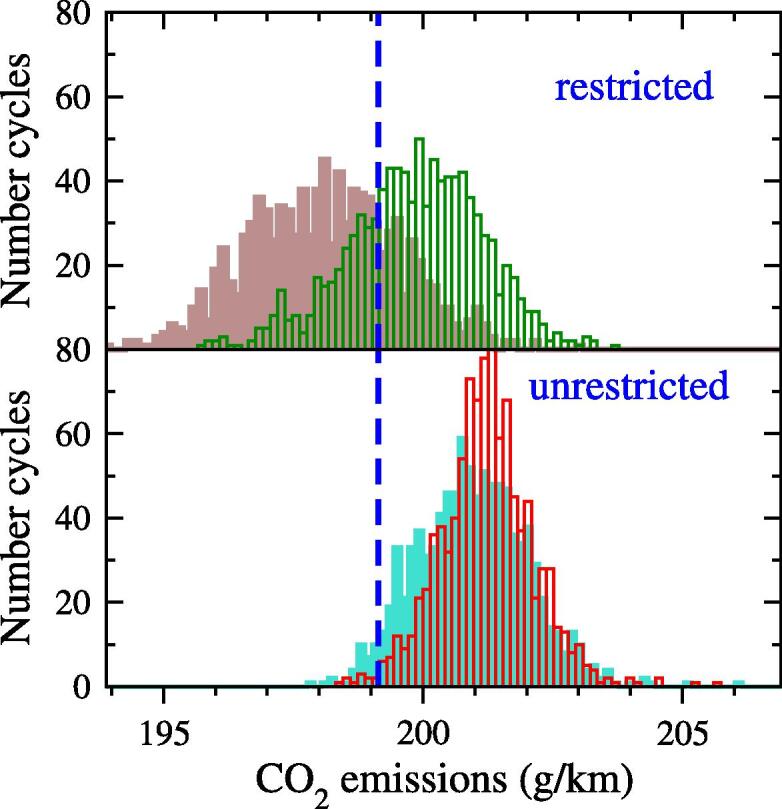
Histograms representing the CO_2_ emissions during the random simulations for the restricted (up) and unrestricted (down) strategies based on the WLTC profile. Empty boxes: Driver 11, Full boxes: Driver 20. Vertical blue-dashed line indicates CO_2_ emissions along WLTC (i.e., TA value). (For interpretation of the references to color in this figure legend, the reader is referred to the web version of this article.)

The CO_2_ distributions predicted by the CO_2_MPAS software following *N_c_* = 1000 random cycles are finally displayed for both drivers in Fig. 14. The figures include the 199.1 g/km of the reference WLTC cycle. The figure shows a similar pattern to the one obtained in Fig. 12. The unrestricted strategy yields CO_2_ distributions with an average value for D11 (199.9 g/km) and D20 (201.2 g/km) that is above the reference WLTC one. For the restricted case, the WLTC emissions fall in-between the two distributions (198.1 g/km and 200.9 g/km in average for each driver). These means differences of < 0.1% with respect to the results obtained using the approximation in 4.2.2.1 (based on direct sampling of fix-IDS cycles), which validates this approach.

## 5 Discussion on CO_2_ emission ranges

**Fig. 15:**
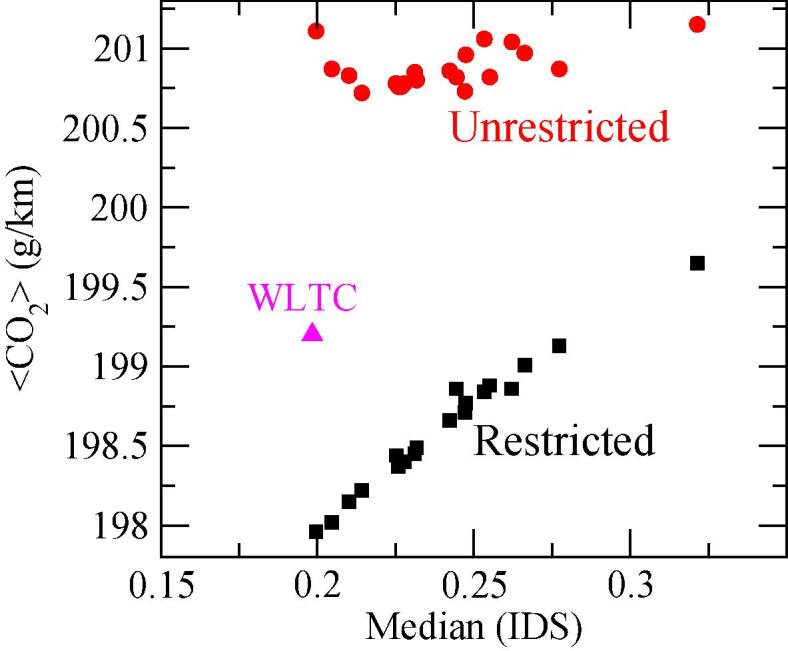
Average CO_2_ emissions versus median IDS for each of the 20 drivers considered. Black squares: Restricted acceleration strategy. Red bullets: Unrestricted acceleration strategy. WLTC correlation point is also shown with a pink triangle. (For interpretation of the references to color in this figure legend, the reader is referred to the web version of this article.)

The results so far indicate a certain dependence of the CO_2_ emissions on the aggressiveness of the driving style. Fig. 15 illustrates this relationship by plotting the average CO_2_ emissions along the average value of the IDS distribution for each driver. There is a clear correlation between both magnitudes when the accelerations are restricted, as a result of the quasi-linear relationship between IDS and CO_2_ values in Fig. 11. On the other hand, the correlation is not clear for the unrestricted case, althouth there seems to be a decreasing trend below IDS = 0.22 and a slightly increasing one for IDS values above.

To understand the differences between the two strategies we must consider additional factors on the vehicle performance. In general, there is an interplay between the higher consumption expected at high speeds -due, among other factors, to the road and air resistances- and the loss of power efficiency when the engine works in the low-speed regimes. We can therefore expect the vehicle to perform more efficiently at moderately low accelerations. Considering the link between the acceleration aggressiveness and the IDS metric, this interplay explains the minimum emission found at IDS = 0.2 for the unrestricted acceleration in Fig. 11. However, in the case of a restricted acceleration case, the decay in the speed profile dominates over the mentioned effect, and the CO_2_ emissions decrease with decreasing IDS values.

At this point, it is convenient to recall the implications of these two strategies. The restricted acceleration assumes that something (e.g., a traffic light, another car, a curve in the road) hinders the free acceleration of the driver, something more adapted to urban and rural driving patterns. On the other hand, the unrestricted behavior presumes that the driver can accelerate at her/his own will without any obstacle ahead, which might better represent motorway driving conditions. Both options can be considered as limit cases, and reality lies in an intermediate situation that combines both assumptions. The median IDS value of the WLTC cycle is 0.198, and CO_2_ emissions obtained along this cycle are 199.2 g/km. As expected, this point falls in-between the restricted and the unrestricted approaches in Fig. 15.

In general, the CO_2_MPAS simulated emission values obtained in this work for real-world driving styles are slightly higher than the value predicted from the WLTC type-approval cycle. Furthermore, the results show that the specific driving style assumed by the driver has a direct impact on the energy consumption and the CO_2_ emissions. We have seen in the previous section that, focusing on sustained specific driving styles (constant IDS) for the whole cycle, this range can get up to ca. 5% variation. This variability should be taken with caution, as it represents an extreme case where the stochasticity of the human acceleration behavior is oblived. However, it points out the limits that could hypotetycally be accepted from extreme cases.

The range of variability decreases significantly to 1% when considering the driving style pattern of each driver. The reason is that most of the drivers show in real world a quite similar stochastic behavior that follows the pattern of lognormal functions. In any case, the results point in the same direction as the conclusions of Pavlovic et al. (Pavlovic et al., 2020) on the fuel consumption variances found for drivers in real-world, and the possible link of this variability with the Fuel Consumption Gap. They attributed a variance of the fuel consumption of circa 5% according to the driver's role, larger than what is reported in our study. However, the nature of both approaches is totally different. The empirical work of Pavlovic et al. deduces the impact of the driver from the measured fuel consumption during on-road trips. This impact is in fact a combination of different factors, and not exclusively the acceleration style, and therefore we can expect enhanced differences in the fuel consumption variability. The driving data of each driver might reflect, for instance, differences in trip lengths, road gradients or speed regimes, as well as differences in the gearshifting style.

## 6 Conclusions

This study quantified the impact of the drivers’ heterogeneity on the vehicle’s CO_2_ emissions. The approach is independent of the vehicle considered and at the same time captures the stochasticity of the driver’s behavior. The quantification is performed on the basis of the official test cycle used in the EU and other countries (WLTC) for emissions and energy consumption characterisation. Although it was explicitely designed from real-world driving segments data, the cycle has a fixed speed profile that reproduces mainly average real-world driving conditions. In particular, considering the distinct real world acceleration style of a specific driver in the WLTC cycle would imply to modify accordingly the cycle speed profile, resulting in unknown variations of fuel consumption of CO_2_ emissions with respect to the certified fuel consumption and CO_2_. This paper tried to fill this gap by providing quantitative results on the relation between drivers’ heterogeneity and CO_2_ consumption over WLTC cycle basis. The degree of aggressiveness during a particular acceleration event is assessed by means of the Independing Driving Style (IDS) metric. Assuming enough observations, a distribution of IDS values can be derived for a particular driver. This distribution is considered the driver’s fingerprint. Going backwards, assuming that such a distribution is known, we can stochastically create a synthetic speed profile based on a template speed cycle.

The main hypothesis of this work is that the driver is more prone to show his/her personal driving style during the acceleration phases than during almost steady-speed driving conditions. Furthermore, each driver shows a different acceleration behavior depending on weather, mood, road, and traffic, resulting in a distribution of IDS values. This is a phenomenon that is not necessarily fully captured in a driving cycle of a fixed velocity profile.

At the same time, we have explored the variability range that can be expected from keeping specific sustained-acceleration aggressiveness, or what can be considered as extreme cases. As a result, we have been able to establish a correlation between a specific acceleration aggressiveness (specific IDS value) and the resulting CO_2_ emissions. Based on the results, we derive the following conclusions:

• The WLTC shows a variable driving behavior. The acceleration style of the 20 drivers in the experimental campaign seems to be more aggressive than the WLTC one. Consequently, the WLTC-benchmarked CO_2_ emissions for these drivers are also above the reference WLTC ones.
• In general, a more aggressive acceleration style can be associated with higher CO_2_ emissions. Nevertheless, this effect might be dimmed in some cases by a decrease in the efficiency at low engine powers.
• The variance that can be expected between the extreme cases in sustained acceleration aggressiveness (defined by maximum and minimum fixed-IDS values) is around 5% of the absolute value in the unrestricted acceleration strategy, and slightly lower for the restricted one.
• The differences in the CO_2_ emissions among the 20 drivers of the real-world campaign are in the range of ca. 2 g/km (ca. 1%), showing a strong correlation between the average values of the IDS and CO_2_ distributions for each driver.

Certain assumptions and aspects of the study have not been covered, such as a clearer distinction of real-world car-following and free-flow acceleration patterns, and the possible roles of gear selection and road gradients. These factors will be investigated in future iterations of the study.

### CRediT authorship contribution statement

The authors, Jaime Suarez Corujo (JSC), Michail Makridis (MM), Katerina Anesiadou (KA),(Dimitrios Komnos (DK), Biagio Ciuffo (BC), and Georgios Fontaras (GF) contributed to the paper as follows: Study conceptualisation: GF, JSC, MM; Methodology: JSC, MM, GF, BC, KA, data collection, curation, and analysis: JSC, DK, BC, KA; model simulations: JSC, DK; analysis and interpretation of results: JSC, MM, GF; manuscript preparation: JSC; revision and comments: MM, BC, GF; research coordination: GF.

## Declaration of Competing Interest

The authors declare that they have no known competing financial interests or personal relationships that could have appeared to influence the work reported in this paper.

## Data Availability

Data will be made available on request.
